# Role of Cervical Spinal Magnetic Stimulation in Improving Posture and Functional Ambulation of Patients with Relapsing Remitting Multiple Sclerosis

**DOI:** 10.1155/2022/6009104

**Published:** 2022-11-21

**Authors:** Shereen I. Fawaz, Shin-Ichi Izumi, Soha M. Hamada, Abir A. Omara, Ghada O. Wassef, Heba Gamal Saber, Sherihan M. Salama

**Affiliations:** ^1^Physical Medicine, Rheumatology and Rehabilitation, Ain Shams University, Egypt; ^2^Physical Medicine and Rehabilitation, Tohoku University, Sendai, Japan; ^3^Audiovestibular Medicine, Hearing and Speech Institute, Egypt; ^4^Public Health, Ain Shams University, Egypt; ^5^Geriatrics and Gerontology, Ain Shams University, Egypt

## Abstract

Balance impairment is one of the hallmarks of early MS. Proprioceptive deficit was found to be one of the main causes of this imbalance. The cervical enlargement has a strong proprioceptive system, with its projections to the reticular formation and the central pattern generators, helping in rhythmic pattern generation and alternate leg movements. Repetitive trans-spinal magnetic stimulation (rTSMS) is a noninvasive technique, which can trigger massive proprioceptive afferents. Therefore, it has the potential of improving proprioceptive deficits and motor control. *Objective*. To determine the effectiveness of repetitive cervical magnetic stimulation in improving functional ambulation of patients with relapsing remitting multiple sclerosis (RRMS). *Design*. Prospective sequential clinical trial. *Setting*. University and academic hospital. *Participants*. A total of 32 participants (*N* = 32) with RRMS. *Interventions*. Outpatient rehabilitation. The 32 patients received 10 sessions over two weeks of 20 Hz cervical spinal magnetic stimulation (SMS). Both groups were assessed at baseline, after 2 weeks, then one month later. Patients were enrolled as a control group at first and received Sham SMS, and then a wash out period of one month was done for all the patients, followed by a baseline assessment. Second, the same 32 patients rejoined as the active group, which received real magnetic stimulation. Both groups performed an intensive physical therapy program with the spinal magnetic stimulation. *Main Outcome Measures*. Extended Disability status score (EDSS), Timed up and Go test (TUG), Mini-Best test, dynamic posturography sensory organization composite score, and motor composite score. *Results*. Thirty-two RRMS patients with EDSS range from 1.5 to 6. They showed statistically significant difference between active and control groups in Mini-Best test score. We divided our patients according to EDSS into 3 subgroups: (a) mild: ≤2.5, (b) moderate: 3-5.5, and (c) severe: ≥6. Mild cases showed significant differences in EDSS score, TUG test, Mini-Best test, and dynamic posturography sensory composite scale. The effect size between the different patient subgroups was also measured and showed highly significant improvements in all measured parameters among our mild patients, indicating that this subgroup could be the best responders to cervical repetitive high-frequency magnetic stimulation. Moderate cases showed highly significant improvement in TUG score and Mini-Best test and significant change in EDSS score and the dynamic posturography sensory composite score. Severe cases showed only significant improvements in TUG, Mini-Best test, and sensory composite score. *Conclusion*. Cervical repetitive magnetic stimulation can help improve balance and functional ambulation and decreases the risk of falls in RRMS patients, especially in the mild, low disability cases.

## 1. Introduction

Multiple sclerosis (MS) is a chronic, inflammatory, demyelinating, and immune-mediated disorder affecting the central nervous system [1]. It is one of the most common causes of disability in young adults secondary to a neurological disease [2]. A recent survey about the epidemiology of MS stated that in 2020, there was a 30% rise in the incidence of MS in comparison to the 2013 incidence rate, with a global prevalence of 35.9 per 100 000 people in 2020 [3]. Balance impairments are the early hallmark of MS even in patients with minimal and early disease status [4–6]. Several factors affect the balance of patients with MS including proprioception, vestibular and cerebellar function, vision, and/or motor weakness [6]. Recent studies suggested that proprioception and postural control deficits are major contributing factors to the increased risk of falls in people with MS, even the mild cases [1, 6–13].

The cervical enlargement of the spinal cord has major contributions to locomotor activity, and it includes a large population of interneurons that mediate coordination and rhythmic regeneration of movements [14]. The proprioceptive system in the cervical region involves short and long axon propriospinal neurons. The short axon neurons referred to as premotor neurons, mediate corticospinal, and sensory input to the upper/forelimb [15]. While the long axon networks project fibers interconnecting the cervical and lumbosacral enlargements through ascending and descending propriospinal tract projections. These networks help modulate input to lower/hindlimb motor neuron pools [16]. The presence of commissural propriospinal connections connects interneurons in laminae 3-4 of the dorsal horn on each side of the spinal cord [17]. Defects in proprioceptive input lead to loss of interjoint limb coordination, hindering its ability to adapt to locomotor behaviors when faced with uneven terrains [18]. Proprioceptive sensory neurons relay information from the muscle spindle and the Golgi tendon organs delivering this information from the muscle and joints, connecting several neuronal subtypes including spinal motor neurons (MNs), local circuit interneurons, and ascending projection neurons [19].

In addition, through propriospinal connections between the cervical and lumbar enlargements, the spinal locomotor pattern generators and central pattern generators (CPGs), which organize neuronal circuits through pattern formation and rhythmic regeneration, help in both initiation and modulation of postural muscle tone, with a major role in functional recovery following spinal cord injury [20]. These pattern formation interneurons help in creating alternating rhythmic locomotor activity, through its excitatory and inhibitory interneuronal systems. Hence, they are intimately involved in the control, synchronization, and coordination of flexor and extensor motor neurons (MNs) of the shoulder/forelimb and pelvic girdle/hindlimb regions, respectively [14, 21].

Motor control is furtherly enhanced by ascending projection pathways from propriospinal interneurons in the cervical region which relay information about the muscle contractile state to the lateral reticular nucleus. In his studies, Takakusaki et al. pointed out that postural control and regulation of locomotion largely depend on projections from the pontomedullary reticulospinal system which largely terminates in the intermediate grey matter of C5/6 and/or C7/8 [22]. These reticulospinal tracts activate neuronal circuits in the cervical part of the spinal cord generating locomotor rhythm through the interneuronal central pattern generators [23]. The rostral spinocerebellar and cuneocerebellar tracts also relay information from the cervical propriospinal to terminate as mossy fibers, forming a major source of input to the cerebellar granule cells [24]. Other pathways which relay information from the cervical propriospinal system are the vestibulospinal tracts which are involved in bilateral locomotion control and postural muscle tone. The spinoolivary tracts synapse neurons from the primary olivary nucleus and inferior olivary nucleus, originating from medial parts of the nucleus properties and the central cervical nucleus and terminates as climbing fibers to the Purkinje cells in the cerebral cortex [25]. These later tracts may be important in the control of movement from the body and limbs. Other subpopulations are the rubrospinal neurons which terminate in the cervical enlargement, with long propriospinal neurons connecting all these axons with the lumbar enlargement, hence, modulating bilateral control of locomotion and posture [14, 15]. Proprioception is also activated by stimulation of muscle vibration, resulting in selective activation of muscle spindle receptor inducing excitation of the primary nerve endings, causing a train of action potentials in afferent large diameter fibers [26].

Spinal cord stimulation (SCS), though an invasive technique, proved through several studies that it can enhance locomotor recovery, through afferent nerve stimulation, recruitment of the motor neuron pool, and activation of ascending pathways projecting to higher brain centers such as the thalamic nuclei, cerebral cortex, and brainstem nuclei. In addition, it induces focal activation of spinal interneurons and central pattern generators, with its role in pattern formation and rhythmic regeneration for locomotor recovery [27].

Repetitive trans-spinal magnetic stimulation (rTSMS) has the advantage of being a noninvasive, remarkably less painful technique in comparison to electric stimulation [28]. Since the underlying mechanism of action of TMS involves the production of an induced electric current from a changing magnetic field, it can trigger massive proprioceptive afferents with minimal activation of cutaneous receptors, together with direct activation of sensorimotor nerve fibers [29]. Similar to SCS, it can stimulate afferent nerve fibers and induce excitation of descending motor pathways. In addition, it can stimulate directly the efferent pathways [30]. Several studies applied rTSMS over the lumbar and thoracic regions and proved that it has a role in reducing spasticity and in pain relief. It also improves motor recovery through activation of local intrinsic circuitry and ascending projections to supraspinal centers [31–33]. In his study, Chalfouh et al. used focal rTSMS over the thoracic region in rat models for the treatment of chronic spinal cord injuries and proved that it can have neuroprotective and neurodegenerative effects. It regulates functional protein synthesis, inhibits demyelination, and enhances neuronal survival and axonal regrowth [34]. Hunanyan et al. also proved that stimulation over the thoracic and lumbar region can induce glutaminergic neurotransmitter release and activate synaptic input to the motor neuron pool resulting in long-lasting facilitation of synaptic transmission to the lumbar motor neurons [35]. Several studies have reported impaired postural control in patients with chronic neck pain of different etiologies [36–38]. Also recently, researchers have focused on balance impairments, proprioceptive deficits, and the increased risk of falls in patients with MS, even in those with low disabilities [1, 6, 39–41]. These studies explained the role of the proprioceptive system and its pathway in balance impairments in MS, even in those with low disabilities. Given the intensive existence of proprioceptive tracts in the cervical region with its connections, both on the spinal and supraspinal level, our study applied trans-spinal magnetic stimulation over the cervical enlargement, aiming to determine its therapeutic effect on postural control and functional ambulation of patients with MS.

## 2. Patients and Methods

The study is a prospective sequential clinical trial that included 32 patients with relapsing remitting MS. Patients were selected from the outpatient clinics of the Physical Medicine, Rheumatology, and rehabilitation department of Ain Shams University Hospitals over the period between 2019 and 2021 and volunteered to join the research. An informed consent was taken from all cases. Patients were diagnosed using McDonald's criteria in 2016 [42]. Included patients were all those aged ≥18 years, and all ambulatory patients with or without an assistive device, with Expanded Disability Status Scale (EDSS), score between 1 and 6 [43]. Only patients in the “remitting” phase were included. Patients excluded from this study were those with disabling medical diseases as severe or recent heart disease and severe head trauma and cognitive impairment: <24 points by minimental scale or uncooperative patients [44]. Patients with any other comorbidity that affects balance and/or posterior column function were also excluded, as well as patients with fixed contractures in the lower limbs, and any contraindications for spinal MS: metal (implants) in the cervical region from welding or metalwork, implanted devices (as spinal cord stimulator, cardiac pacemaker, and cochlear implants), and pregnancy. The study protocol was approved by the Ethics Committee of the Faculty of Medicine, Ain Shams University. All the assessments were done by blinded physicians except the first author, and all the physical therapy was done by a well-experienced blinded therapist in applying rTSMS.

The sample size was calculated using NCSS PASS 11.0 and based on a pilot study that was performed before carrying out the original research work as we extensively reviewed the current literature, and no previous studies were found to address the present study objectives. Sample sizes of 32 patients would achieve 99% power to detect a difference of 1.53 between the group means (the dynamic posturography sensory composite score) with standard deviations of 1.03 and 0.59 at a significance level (alpha) of 0.005000 using a two-sided *z*-test. These results assume that 5 sequential tests are made using the O'Brien-Fleming spending function to determine the test boundaries. Enrollment of all 32 patients was done as their own control group at first, where all patients received Sham SMS for 2 weeks (5 sessions/week of 20 Hz cervical spinal magnetic stimulation (SMS); total 10 sessions over two weeks), followed by reassessment 1 month afterward. A washout period of one month was done for all the patients, followed by a baseline assessment afterwards to rejoin as the active group, which received real magnetic stimulation, for 2 weeks followed by reassessment at the end of the protocol, then 1 month later afterwards. All patients performed an intensive physical therapy program with the magnetic stimulation.

## 3. Magnetic Stimulation

Magnetic stimulation was done using Neurosoft equipment (Neuro-MS/D Variant-2 therapeutic Neurosoft, Russia). A circular coil was used for cervical spinal nerve root stimulation; the lower margin of the coil was applied at the level of C7 cervical vertebrae, about 2 cm paravertebrally, and oriented with the grip vertical to the spinal cord; this is a good coil position for stimulating spinal nerve roots. The coil generated a magnetic field of up to 2 Tesla at the periphery of the coil.

### 3.1. Study Tools

Full medical history was taken from all patients, and clinical examination including full neurological examination was also done. We divided our patients according to the Expanded Disability Status Scale (EDSS): (a) mild disability: cases with EDSS scores less or equal to 2.5, (b) moderate disability cases with EDSS equal 3-5.5, (c) severe cases with EDSS score equal to or more than 6. Primary outcome measures included Functional scores as the Timed up and Go test (TUG) test [45] and Mini-Best test, with a maximum score of 32 [46]. Computerized dynamic posturography [47] was done with the measurement of sensory organization composite score and motor composite score. Secondary outcome measures were MRC scale [48] to major muscle groups in both lower limbs (iliopsoas, quadriceps, ankle dorsiflexors, and hip abductors) and Modified Ashworth Scale [49] to both lower limb muscles (adductors, knee extensors and flexors, and ankle flexors and extensors).

## 4. The Rehabilitation Protocol

Both groups received 10 sessions over two weeks of therapy. The active group received real rTSMS frequency of 20 Hz, for 5-second work period, with intertrain interval of 10 seconds. The number of trains is 30. With a total number of 3000 pulses per session. The intensity is at 35% of Maximal Stimulator Output. The control group received Sham rTSMS sessions, using the specialized program for placebo trials. The physical therapy program was done for 45 minutes; in the form of trunk abdominal strengthening exercises, static and graduated-resisted exercises were done for both upper and lower limb strengthening and stretching exercises and intensive balance training exercises.

## 5. Data Management and Statistical Analysis

The collected data was revised, coded, tabulated, and introduced to a PC using a statistical package for social sciences (IBM SPSS 20.0). Data were presented, and suitable analysis was done according to the type of data obtained for each parameter. Mean and standard deviation (+ SD) range for parametric numerical data, and 95% CI was calculated for each parameter. An independent sample *t*-test was performed to test for statistically significant difference between mean change in two independent groups. *P* value: level of significance; *P* > 0.05: nonsignificant (NS); *P* < 0.05: significant (S); *P* < 0.01: highly significant (HS).

## 6. Results

Our study included 19 males and 13 females. Their ages varied between 19 and 52, with a mean of 35.81 + /−9.12 SD. There was highly significant improvement (*P* value <0.001) in the total EDSS, TUG scores, Mini-Best test, and the dynamic posturography test (both motor and sensory composite scores) in the active group but not in the control group. All cases nearly maintained the same values one month afterwards.

**Figure 1:**
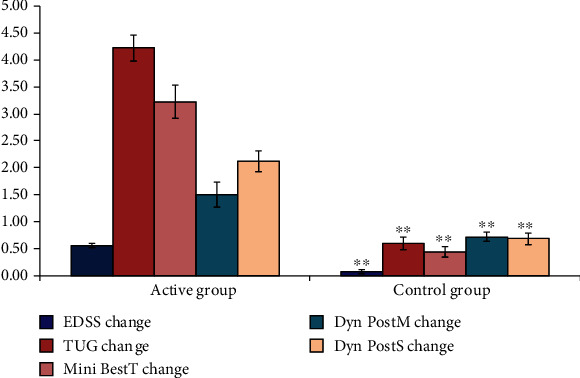
Comparison between active and control as regards to all studied variables. SD: standard deviation; ^∗^*P* < 0.05 between group comparison; ^∗∗^*P* < 0.01.

In an attempt to find the best responders to cervical SMS, we determined the effect size, by measuring the amount of change produced by rTSMS postrehabilitation from the initial parameters of the patient. There was a statistically highly significant difference change (*P* value 0.000) between active group and control group as regards to the change in each of EDSS, TUG change, Mini-Best test, dynamic posturography sensory organization score (DPSOS), and significant change in dynamic posturography motor composite score (DPMCS) (*P* value 0.003) (Figure 1).

**Figure 2:**
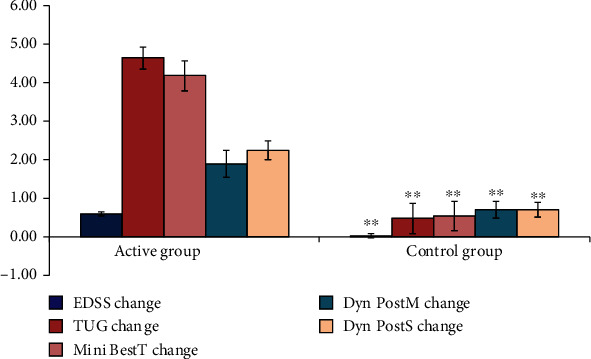
Comparison between mild active group and mild control group as regard to all the studied variables. SD: standard deviation; ^∗^*P* < 0.05 between group comparison; ^∗∗^*P* < 0.01.

Regarding the patients' subgroups, the group with mild low disability gave the best results, with a statistically highly significant difference change (*P* value 0.000) between active group and control group as regards to the change in EDSS, TUG, Mini-Best test, DPSOS, and significant change in DPMCS (*P* value 0.005), Figure 2).

**Figure 3:**
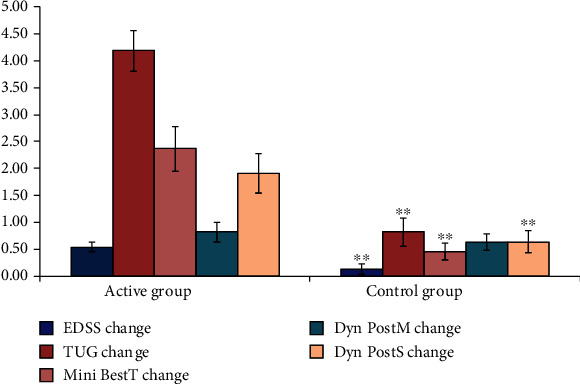
Comparison between moderate active group and moderate control group as regard to all the studied variables. SD: standard deviation; ^∗^*P* < 0.05 between group comparison; ^∗∗^*P* < 0.01.

The moderate group was slightly lower than the mild cases, and there was a statistically highly significant difference (*P* value 0.000) as regards to the change in Mini-Best test and TUG scale. Significant changes were also found in the change of the EDSS (*P* value 0.004) and DPSCS (*P* value 0.008). While the DPMCS did not show significant difference (Figure 3).

**Figure 4:**
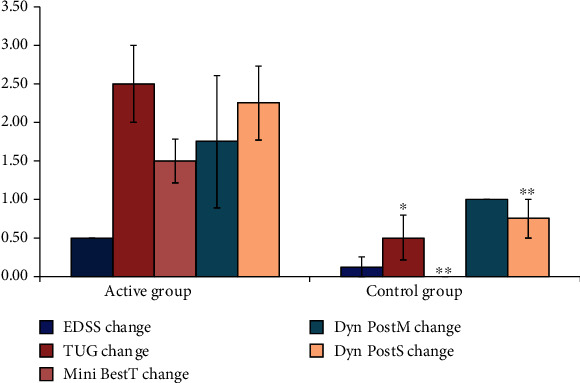
Comparison between severe active group and severe control group as regard to all the studied variables. SD: standard deviation; ^∗^*P* < 0.05 between group comparison; ^∗∗^*P* < 0.01.

In accordance, the severe active cases showed less improvement compared to the other subgroups. However, there was still a statistically significant difference as regard TUG change (*P* value 0.013), Mini-Best test (*P* value 0.002), and DPSCS (*P* value 0.032). Changes in EDSS and DPMCS did not show significant differences (Figure 4). There was no significant change in the secondary outcome measures, both the MRC scale (*P* value 0.113) and the Modified Ashworth Scale (*P* value 0.239), between pre- and postrehabilitation protocols. Adverse events included only pain in thin patients. The pain did not require to holt the treatment or postpone the sessions. The intensity was lowered by 2-5% to control the pain. Unintended effects were the patient reported improvement in the writing and fine movement skills.

## 7. Discussion

This study supports the concept that cervical stimulation can help improve balance impairments in patients with RRMS. Recognizing the proprioceptive deficits that are associated with the imbalance and increased risk of falls in patients with RRMS, our study showed a significant improvement in the Mini-Best test over other variables when comparing the total active and total control groups. This goes in agreement with the work of Fling et al. that balance impairment in RRMS patients is largely based on defects in the proprioceptive pathway [1].

In addition, when dividing our patients into three categories and comparing the two groups, mild cases in the active group showed highly significant differences in EDSS score, TUG test, and Mini-Best test, and significant difference in the dynamic posturography sensory composite scale which supports the idea that cervical stimulation could be more effective in balance rehabilitation, especially in this subpopulation group. This supports the work of other studies pointing to the early impairments of the somatosensory system in even low disability RRMS patients [6]. The observed improvements upon cervical magnetic stimulation could be attributed to the higher number of residual long descending projections to the lumbar enlargements of the spinal cord, which helps coordination of interlimb movements. In addition to the ascending connection with both the reticular formation and the cerebellum, it may contribute to the enhanced motor control, together with associated stimulation of the motor neurons in the cervical region and muscle spindle activation through muscle contractions.

While moderate EDSS-scored patients showed near significant improvement in the Mini-Best test, and severe cases showed significant change in the Mini-Best test. This could be supported by the high sensitivity of the Mini-Best test over other functional scores, such as the TUG test [50] and its reliability in assessing balance and its change over time [51]. Other measurable outcomes in both subgroups showed nonsignificant improvements.

Our study also showed highly significant improvement in determining the effect size, measured as the change between postintervention and the initial parameters in the different patient subgroups. Patients defined as mild showed the highest significant responses in both the TUG test and Mini-Best test and the sensory and motor composite score of the dynamic posturography test. This shows that this population group could be the best responders to cervical stimulation resulting in activation of propriospinal connections, ascending and descending, and enhancing locomotion, balance, and functional ambulation, especially in low disability MS patients.

Accordingly, the other two groups show improvement, with patients with 6 or more EDSS scores showing fewer benefits from this therapy. The most possible explanation is that the associated pyramidal tract affection and/or other systems need other associated therapies that would specifically target them, as cortical, cerebellar, or even neuromuscular peripheral stimulation, combining neurorehabilitative approaches might be essential in those cases.

Several studies applied high-frequency spinal magnetic stimulation to reduce spasticity using lumbar magnetic stimulation and showed significant reduction in spasticity, and they speculated that these results could be due to an increase in proprioceptive input produced by the magnetic stimulation to the spinal motor system and its supraspinal control centers [32, 52]. While Nielsen et al. used high-frequency magnetic stimulation over the midthoracic region and showed reduction in spasticity and an increase in voluntary motor power [53], explaining their results is the massive activation of the peripheral nerves; ventral nerve roots exciting the deep-seated motoneurons. The spinal motor neurons activate long loop pathways which include the propriospinal pathway, connecting the cervical and lumbar enlargements, coordinating interlimb movements, and the spinobulbospinal pathways. In addition, the induced current from the magnetic stimulation causes direct activation of intraspinal axons with neuronal spinal excitation. They also explained the reduction in spasticity due to an increase in Ia afferent input that is mainly mediated by the propriospinal pathways to the motor neuron pool [33, 53, 54]. Therefore, as proven by the work of several studies on both the lumbar and thoracic regions, spinal magnetic stimulation can have a significant role in the activation of the long propriospinal pathways connecting the cervical and lumbar enlargements with its influence in contributing to the reduction of spasticity and enhancing limb coordination and motor control. Our study applied the SMS over the cervical region aiming to activate the intensively located propriospinal connections in this region to increase postural control and locomotion in our patients.

## 8. Conclusion

**Figure 5:**
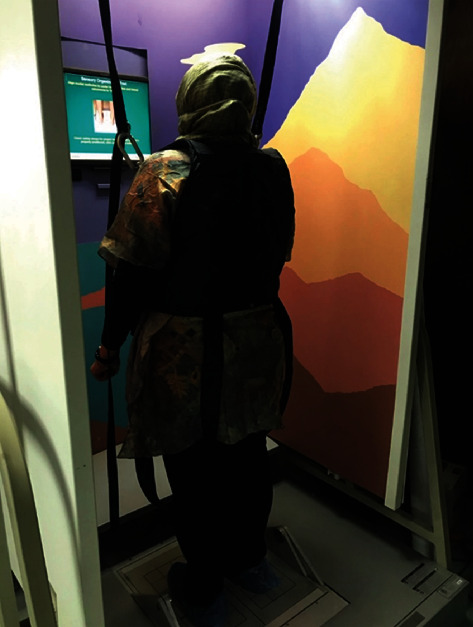
Patient on dynamic posturography test.

To our knowledge, studying the role of cervical magnetic stimulation as regards to its therapeutic effect on improving proprioception, postural control, and functional ambulation is still relatively novel. The suggested mechanism of action is related to the specific role of the cervical propriospinal, being privileged with its connection with the reticulospinal tracts, together with rubrospinal, vestibule, and spinal tracts, along with their role in enhancing motor control [52]. Comparing the effect of cervical and lumbar enlargements, in improving balance dysfunction in RRMS, patients will still need to be studied further (Figure 5). The limitation of this study is the short treatment time. It is recommended to prolong the treatment duration to 3 or 4 weeks followed by maintenance therapy of average once a week for 3-6 month.

## Data Availability

All data are available upon request.
